# Murine Typhus: Clinical and epidemiological aspects

**Published:** 2012-06-30

**Authors:** Gaspar Peniche Lara, Karla R Dzul-Rosado, Jorge Ernesto Zavala Velázquez, Jorge Zavala-Castro

**Affiliations:** a Universidad Autónoma de Yucatán, México gaspar.peniche@uady.mx; b Unidad Interinstitucional de Investigaciónclínica y Epidemiológica, Facultad de Medicina, zavala@uady.mx; c Centro de Investigaciónes Regionales Dr Hideyo Noguchi, Facultad de Medicina. zcastro@uady.mx

**Keywords:** *Rickettsia typhi*, *Rickettsia*, infection, rats, fleas, rickettsioses

## Abstract

**Rickettsia typhi:**

is an intracellular bacteria who causes murine typhus. His importance is reflected in the high frequency founding specific antibodies against *Rickettsia typhi* in several worldwide seroepidemiological studies, the seroprevalence ranging between 3-36%. Natural reservoirs of *R. typhi* are rats (some species belonging the *Rattus* Genus) and fleas (*Xenopsylla cheopis*) are his vector. This infection is associated with overcrowding, pollution and poor hygiene. Typically presents fever, headache, rash on trunk and extremities, in some cases may occur organ-specific complications, affecting liver, kidney, lung or brain. Initially the disease is very similar to other diseases, is very common to confuse the murine typhus with Dengue fever, therefore, ignorance of the disease is a factor related to complications or non-specific treatments for the resolution of this infection. This paper presents the most relevant information to consider about the rickettsiosis caused by *Rickettsia typhi. *

## Abstract (es)

**Rickettsia typhi:**

es una bacteria intracelular causante del tifo murino. Su importancia queda reflejada en la elevada frecuencia con que se encuentran anticuerpos específicos frente a *Rickettsia typhi* en diferentes estudios seroepidemiológicos a nivel mundial, variando la seroprevalencia entre el 3-36%. *R. typhi* tiene como reservorios naturales a las ratas (especies del Género *Rattus*) y como vector las pulgas (*Xenopsylla cheopis*). Esta infección está asociada comúnmente al hacinamiento, contaminación y falta de higiene. Clínicamente se presenta fiebres, cefalea, exantema en tronco y extremidades, en algunos casos pueden presentarse complicaciones órgano-específicas, afectando hígado, riñón, pulmón o cerebro. Inicialmente la enfermedad es muy similar a otras enfermedades, siendo muy común confundir al tifo murino con fiebre causada por Dengue, por lo tanto, el desconocimiento de la enfermedad es un factor relacionado a complicaciones ó tratamientos poco específicos para la resolución de esta infección. Este trabajo presenta la información más relevante a considerar sobre la Rickettsiosis causada por *Rickettsia typhi*.

## Introducción

Bacteria belonging *Rickettsia* Genus are intracelular obligate organisms, gram negative with ability to infect arthropods like fleas, ticks as well as small vertebrates.

Initially, bacteria from *Rickettsia* Genus have been grouped, based on their clinical manifestation, immunological reactivity, intracellular localization and G+C amount on his DNA in two groups: Tifus group (TG) and Spotted Fever Group (SFG). Phylogenetic evaluation based comparing 16RNAe gene, have been proved that Rickettsia belongs to Proteobacteria class sub group^1^. Complete genome analysis from several *Rickettsia* species actually propose a new division in four rickettsial groups: Tifus group (*Rickettsia typhi* y *Ricketsia prowazekii*); Spotted fever Group (*Rickettsia conorii*, *Rickettsia sibirica*, *Rickettsia rickettsii*); Ancestral Group (*Rickettsia canadensis *y *Rickettsia bellii*) and transition Group (*Rickettsia felis* y *Rickettsia akari*)^2^.

This study will focus about infection caused by *Rickettsia typhi*, *Rickettsia* specie that belongs to Tifus Group who causes murine tifus *Rickettsia typhi* was identified in 1928 by Dr. Hermann Mooser, Dr. Maximiliano Ruiz Castañeda and Dr. Hans Zinsser in Mexico studying the so-called ''Mexican typhus'' because of the similarity in symptoms with the exantemic typhus caused by *Rickettsia prowazekii*, reporting that this disease, contrary to exantemic typhus, which is transmitted by the louse is transmitted by rats and their fleas species will detail later. Initially, this *Rickettsia* was called like his discoverer: Hermann Mooser so the initial name was *Rickettsia mooserrii* ^3^. Subsequently, this bacteria was identified in others continents considering as a bacteria with a worldwide distribution (Table 1).

**Table 1:**
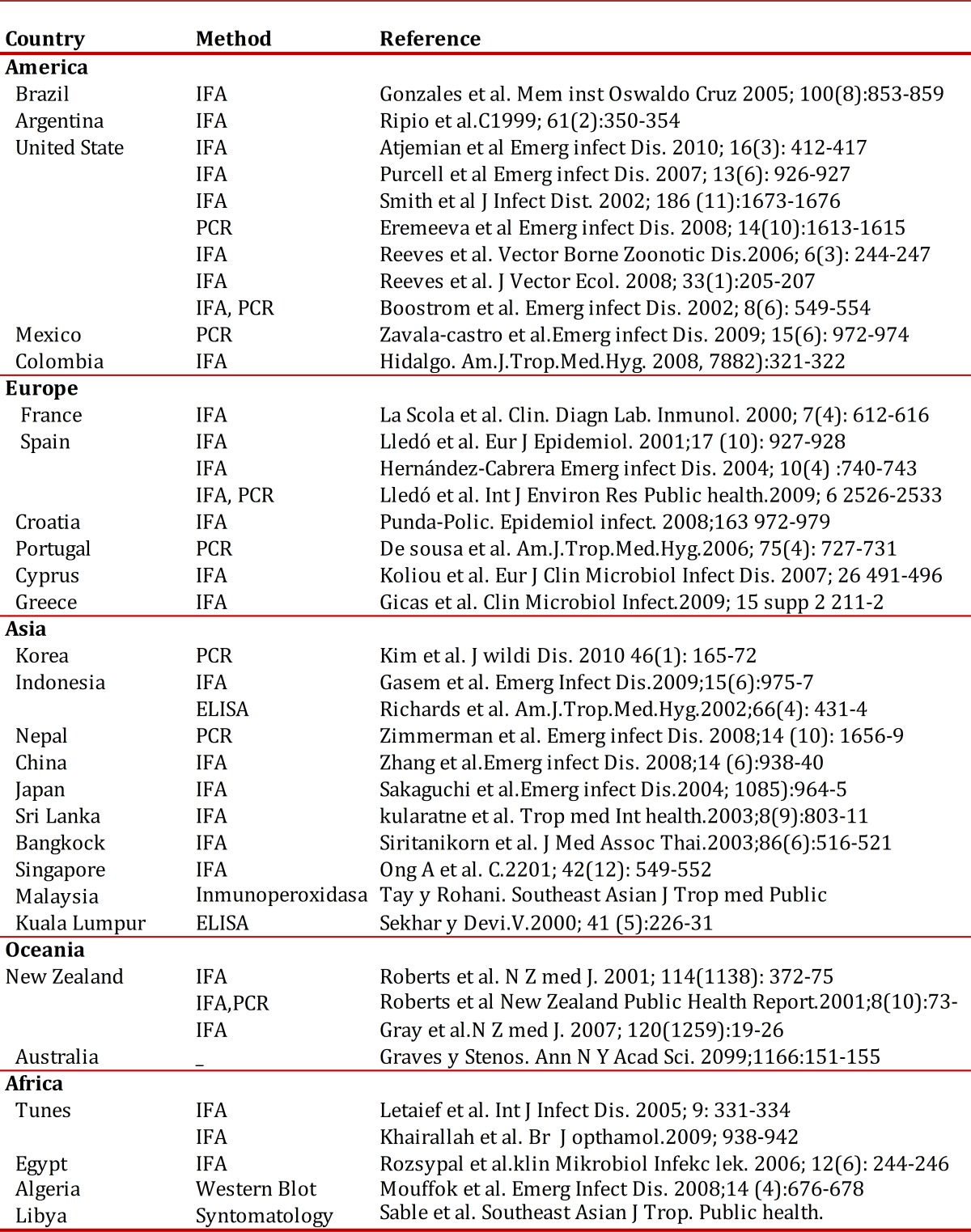
Rickettsia typhi reports in the XXI century

## Causal agent

*Rickettsia typhi* as well as *Rickettsia prowazekii*, belongs to Tifus Group in the Rickettsiaceae Family from Rickettsialis Order and is the causative organism of murine or endemic typhus^2^. Actually, infections with *Rickettsia* *felis* are considered as a murine typhus due to similarity in symptoms with murine typhus. This causal agents, share common characteristic from all the *Rickettsia *species. Both are genetically similar, his classification was based on cell surface protein characterization (OmpA and OmpB) and lipopolysaccharides (LPS); due to both groups have the 17 kDa protein, lipopolysaccharides and OmpB but, unlike *Rickettsia typhi*, *Rickettsia felis* have an additional outer membrane protein OmpA^2^ this is why initially *R*. *felis* was considered a Spotted Fever Group *Rickettsia*. To date, *R. felis* share characteristics from both groups are considered as a *Rickettsia* belonging to the transition Group^2^.

Both bacterias a located in celular cytoplasm at the infection time, having the characteristic of freedom from the vacuole formed when *Rickettsia* enter to the cell by induced phagocytosis by the same *Rickettsia* ^3^

##  Rickettsia typhi life cycle

**Figure 1:**
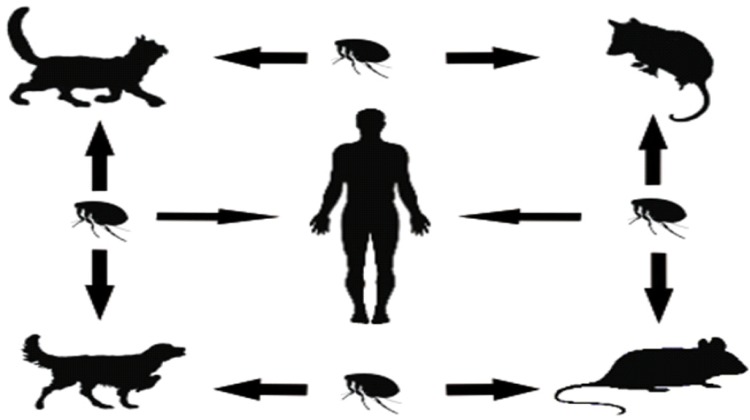
Biologic Cycle of Rickettsia typhi

This cycle are composed by mammals host (rats and humans) and vectors (fleas). The classic natural cycle of this agent includes as a reservoirs two rats species (*Rattus rattus* and *Rattus norvergicus*) and the flea *Xenopsilla cheopsis* as a vector (Fig. 1). The fleas acquire the infection from rats with rickettsemia maintaining the infection during all his life but not killing the vector. Infection in humans are acquire in three different ways, being the most frequent way he self-inoculation from feces of fleas in the bite area and nails, this due to the presence of fleas in skin which produces itching that leads to the itching. Other transmission way includes bite and inhalation of flea infected feces when the hygienic conditions are inappropiated ^4^. This classic cycle is still the main cause of endemic typhus in some regions in Greece and United States^5^. In other areas, murine typhus have other patterns not characterized. The main aspect is the presence of others reservoirs (i. e. cats, dogs or opossums), other vector and many others *Rickettsia* species^6^. In United States, contrary to the classic cycle rat-flea-rat, the most important reservoirs are opossums from the gender *Dydelphis* and cats¸ the cat flea, *Ctenocephalides felis* also have been identified as a vector^4^.

## Pathogeny

Results obtained about endemic typhus pathogeny are mainly based *in vitro* studies.

Rickettsial pathogeny depends of intracythoplasmatic niche rich in nutrients and grows requirements inside the cell host. Invasion to cell is an essential previous requirement for intracellular replication and after all intracellular diffusion.

After the entry of the organism through the skin or the respiratory system spread via the lymphatic and/or blood to the endothelial cells that are its main target. Endothelial injury is the key element in the pathogenic and pathophysiology of endemic typhus. *R. typhi *adheres to endothelial cells through outer membrane proteins. Among the major outer membrane surface proteins are OmpA and OmpB which are present in the Rickettsial Spotted Fever Group and the Transition Group, while the Typhus Group *Rickettsia* only have OmpB and his cellular receptor still unknown. Although, initial OmpA inhibition studies, identified as a protein critical for *R. rickettsii* adhesion to host cells^7^, recent studies based on proteomic analysis has revealed two new alleged Rickettsial adhesins, one of which is the C-terminal peptide of β rOmpB and the other is encoded by the gene RC1281 in *R. conorii* and RP828 gene in *R. prowazekii* ^8^. Interestingly, OmpB interacts with Ku70 a predominance of nuclear DNA-dependent of protein kinase, which is also present in the cytoplasm and plasma membrane, and this interaction has been implicated in the internalization *R. conorii* in Vero cells and HeLa Cells. Immediately to his adhesion, *R. typhi *penetrate endothelial cells by phagocytosis induced by the pathogen. Rickettsial invasion requires the presence of cholesterol-rich microdomains containing Ku70 and the ubiquitin ligase, c-CBL, the input focus to the ubiquitination of Ku70^9^.

There is additional evidence for possible involvement coordinated upstream through the signaling mechanisms Cdc42 (a GTPase), phosphoinositide 3-kinase, c-Src and other tyrosine kinases in the activation of pathways Arp2/3 complex or other. However, activation of p38 MAPK suggests a role for actin polymerization in host cell internalization *Rickettsia* ^16^ ^,^ ^17^. this way, recent evidence also suggests that Ku70-rOmpB interactions are sufficient to mediate invasion of host cells and *Rickettsia* non phagocytic internationalization process also includes contributions to endocytosis via clathrin-and caveolin-2-dependent^10^. Recent research with electron microscopy indicate that the entry of *Rickettsia* in mammalian cells occurs within minutes after contact, this interaction, therefore, is almost instantaneous and once internalized, *Rickettsia* is able to escape quickly in the cytoplasm, probably before fusion phage lysosome and is suspected is done through a phospholipase activity^11^.

In fact, phospholipase activity may be responsible for damage to the host cell membrane that occurs during entry and exit of the *Rickettsia* from cells. Once inside, spreads to nearby cells by a peculiar mechanism involving rearrangement of actin and endothelial cell production of direct endothelial injury in which free oxygen radicals are involved^12^.

## Clínical manifestations

Clinical manifestations begin after 7-14 days nonspecifically incubation period; the most common symptoms are fever, musculoskeletal pain, headache and rash. This occurs in 60-70% of cases, usually appears on the fifth day of onset of symptoms and lasts an average of 4 days is usually maculopapular thin, affecting the trunk and extremities and respects the palms and soles. The clinical course in most cases is mild with fever and disappearance of additional symptoms in 10-14 days, the specific treatment defervescence occurs in 2-4 days. The percentage of organ-specific complications (pneumonitis, hepatitis, meningoencephalitis, renal failure) does not usually exceed 10%, and severe cases (development of refractory shock, respiratory distress, multiple organ failure, hemorrhagic diathesis, consumptive coagulopathy, or severe neurological compromise) there are only around 2-4%, mortality of murine typhus ranges from 0-1%. Different factors have been associated with a more severe course of disease, among which are age, the presence of various hematologic diseases (hemoglobinopathies), early laboratory abnormalities such as renal failure, hypoalbuminemia, hyponatremia and hypokalemia, the late start of treatment effective treatment cotrimoxazol^13^.

## Diagnosis

Historically, differentiation between *Rickettsia *species has been carried out by serological and many other methods.

The Weil-Felix test was used in the past as a presumptive test for the identification of rikettsiosis in routine laboratories, is based on the detection of antibodies to various *Proteus* species which contain antigens that cross-react against epitopes of members of the genus *Rickettsia *with the exception of *R. akari* ^14^. However, the low sensitivity and specificity of the Weil-Felix test for diagnosis of RMSF (Rocky Mountain Spotted Fever)^15^, place it as a test of limited relevance to be used in the clinic.

**Figure 2:**
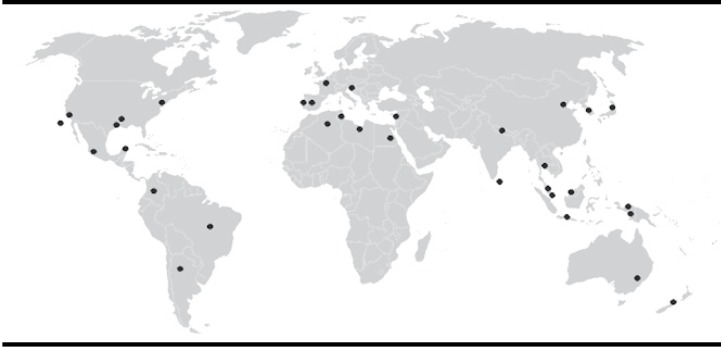
Worldwide distribution of R. typhi in the XXI century

ELISA Test (enzyme immunoassay) was the first to be introduced for the detection of antibodies against *R. typhi *and* R. prowazekii*, the use of this technique is very sensitive and reproducible. This technique allows the differentiation of IgG and IgM, and has been adapted for the diagnosis of RMSF and scrub typhus^16^.

Another serological test hasn't been widely used, is the microagglutination due to the need of large quantities of purified rickettsial antigen and these antigens are not available commercially^16^.

The IFA (immunofluorescence assay) technique is the ''gold standard'' and is used as a reference technique in most research laboratories for serodiagnosis of rickettsiasis, to determine IgG and/or IgM. IFA identification of specific IgM antibodies in several species of Rickettsia provides strong evidence of recent active infection, although the diagnosis may be obscured by a prozone phenomenon and can also be affected by the rheumatoid factor^17^.

The immunoperoxidase assay was developed as an alternative to IFA for the diagnosis of scrub typhus and was later evaluated for use in the diagnosis of infections caused by *R. conorii* and *R. typhi, *the sensitivity and specificity obtained by immunoperoxidase assay for the serodiagnosis of scrub typhus, epidemic typhus, and MSF (Mediterranean spotted fever) is similar to those obtained by IFA^18^. The first proposed method of identification based on molecular biology was the PCR / RFLP method of the gene that encodes citrate synthase, which allowed differentiation of nine species of rickettsiae of SFG. Later, using a combination with a method based on PCR-RFLPs analysis of OmpB gene fragment allowed differentiation of 36 strains of SFG^19^

## Epidemiology of murine typhus

This disease is endemic in temperate climates and especially in coastal areas. In the United States, Asia, Australia, México and Spain (Table 1, Fig. 2). Also have been founded *R. typhi* infection in different species of wild mammals in different parts of the world which can include rodents (*Rattus rattus, Rattus norvergicus*), opossums (*Dydelphis*) and dogs as well as consider endemic typhus as a disease imported by travelers and refugees^20^. It has been shown by studies of incidence of this disease in different countries, which are seasonal, in which the majority of cases occurring in a year is higher during warm weather, while cold weather, infection is very low or almost zero. This disease occurs in all age groups and is relatively common in children. As regards distribution by sex, race and occupation of patient no significant differences, although people living in rural or disadvantaged areas are more prone to infection.

In America, there are records of this disease caused by *Rickettsia typhi* in Mexico since 1928, which, as already mentioned in the introduction, in collaboration with Hermann Mooser, Maximiliano Ruiz Castañeda and Hans Zinsser identify the causative agent of murine typhus or endemic in Mexico^3^. Currently there have been reports of the presence of *Rickettsia typhi* in America in countries like Brazil in 2005, which reports the presence of rickettsial antibodies to *Rickettsia typhi* in a rural community as well as other *Rickettsia* and *Rickettsia rickettsii*, causal agent of Rocky Mountain Spotted Fever^21^; similar study was conducted in Argentina also founding these antibodies in a healthy population of a community rural^22^. The importance about these studies is the presence of *R. typhi* in the population which has already been infected possibly being misdiagnosed.

## Discussion

*Rickettsia typhi *is a common bacteria all over the world, is preferably in warm climates and coastal areas. His wild vectors and reservoirs are very common in most countries. Murine typhus, the disease caused by this bacterium is related through history with famine and overcrowding, with the rural population more susceptible to infection. Today, in Mexico, the knowledge that we have about this disease is very rare because there have been no reports of this infection in our country since the mid-twentieth century, where in central Mexico which subsequently caused epidemics able to control disease was considered eradicated. It was early 2000 when it was detected in a seroprevalence study in the State of Mexico, the presence of antibodies against *R. typhi* and in late 2009 where he reported the first case of *R. typhi* infection in Yucatan State, Mexico by possibly have been filed or are filing cases of infection by *R. typhi* and ignorance of the disease is not diagnosed correctly. In Mexico, medical school curricula listed as a rickettsial disease which is not present in the country which leads to ignorance of the disease and its confusion with a fever caused by Dengue in most cases. A serious strategy to update the curriculum to include rickettsial infection as a health problem in Mexico and possibly other countries. Also, the needs to identify their presence and life cycle not only in Mexico but in the Americas since principally are tropical regions where they might be other vectors of this rickettsial species which unfortunately to be low-income areas, can be a greater likelihood of infection, since it has the geographic and climatic conditions to dwell this bacterium. This study was conducted with the aim of presenting the most complete information about *R. typhi* and the disease it causes to which the Mexican community and the continent is exposed.
